# A comparison of electronic health records at two major Peking University Hospitals in China to United States meaningful use objectives

**DOI:** 10.1186/1472-6947-13-96

**Published:** 2013-08-28

**Authors:** Jianbo Lei, Paulina Sockolow, Pengcheng Guan, Qun Meng, Jiajie Zhang

**Affiliations:** 1 Center for Medical Informatics, Peking University, Beijing, China; 2 School of Biomedical Informatics, University of Texas Health Sciences Center at Houston, Houston, TX, USA; 3 Drexel University College of Nursing and Health Professions, Philadelphia, PA, USA; 4 Center for Statistics and Informatics, Ministry of Health, Beijing, China

**Keywords:** China, Electronic health record, Standard, Meaningful Use, United States

## Abstract

**Background:**

In accordance with the People’s Republic of China’s (China) National Health Reform Plan of 2009, two of the nation’s leading hospitals, located in Beijing, have implemented electronic medical record (EMR) systems from different vendors.

To inform future EMR adoption and policy in China, as well as informatics research in the US, this study compared the United State’s Hospital Meaningful Use (MU) Objectives (phase 1) objectives to the EMR functionality of two early hospital EMR adopters in China.

**Methods:**

At both hospitals, the researchers observed a physician using the EMR and noted MU functionality that was seen and functionality that was not seen yet was available in the EMR. The information technology department was asked about the availability of functionality neither observed nor known to the physician.

**Results and conclusions:**

Approximately half the MU objectives were available in each EMR. Some differences between the EMRs in the study and MU objectives were attributed to operational differences between the health systems and the cultures in the two countries.

## Background

In 2009, the Chinese government released its ambitious secondary round of their national health reform plan. The plan’s overall goals include establishing a sound basic health care system covering urban and rural residents and providing safe, effective, convenient, inexpensive medical and health services [1]. This reform plan includes a framework plan and an implementation plan with the following important features. First, they are improving medical insurance coverage and extending this coverage to 90% of the population. As of 2011, 95.6% of the population had insurance coverage [2]. Second, instead of prescription medications being available solely from hospitals, the government has a plan to establish a medication system of almost 700 essential drugs available from grassroots healthcare providers at government-guided retail prices. Third, the government has a plan to enhance primary care infrastructure by improving the quality of county hospitals, community health centers, and grassroots healthcare professionals. Meanwhile the government also plans to encourage residents to use community health centers as their entry point to the health system instead of hospitals. Lastly, the government will facilitate equal access to public health programs by strengthening community-level programs aimed at selected demographic groups and major diseases [3].

In China, health information technology (HIT) has been recognized as one of the eight supporting pillars necessary to achieve the goals of health reform [1]. Evidence has shown that HIT is essential to improving patient safety and quality of care [4]. HIT is important because it will enable information sharing and efficient interoperability of medical and health information with information systems in public health, health insurance and pharmaceutical industries, and financial regulation related to health care. EHR implementation is also viewed as necessary for modern hospital management, equalization of the distribution of health care services, improvement of efficiency, protection of medical quality and safety, and effective use of limited medical resources. This acknowledgement of the importance of HIT in China’s health reform plan has stimulated policy that supports HIT investment. The Ministry of Health (MOH) has initiated policies to promote HIT. The MOH established a standards bureau office which issued a series of HIT standards. Some of the EHR related standards are listed as follows:

•EMR basic architecture and data standards, released on 7/31/2009 [5]

•EMR Basic Regulations, released on 2/21/2010 [6]

•EMR system Functional Profiles, released on 12/31/2010 [7]

•Technical profiles of EMR-based Hospital Information Platform [8]

•Technical profiles of EHR-based regional Health Information Exchange Platform [9]

It should be noted that in China, EMR and EHR convey distinct meanings (whereas in the US, the two terms are often used interchangeably). In China, EMR refers to systems that manage patient records for clinical purposes; EHR refers to systems that manage longitudinal health data for the population usually stewarded by public health agencies [8].

Financially, the Central Government allocated 9.5 billion RMB (approximately 1.5 billion USD) in 2011 as part of the health reform package to promote use of HIT in China’s hospitals. This investment exceeded the total investment in HIT over the last 30 years. Investments by MOH included a pilot program to implement EMRs in 97 hospitals in 2010 [10] and in an additional 92 hospitals in 2011 [11]. In addition, 16 provincial level trial programs for regional health exchange were supported and all county hospitals in China’s central western provinces were each financed by about 2 million RMB to acquire hardware and software for implementing HIT.

This policy and financial support greatly accelerated the adoption of EHR in China. According to a 2012 national survey in China, nearly 50% of 1,004 responding hospitals had adopted the basic forms of electronic health records (EHR) systems (48.18%), practice management systems- often referred to as Hospital Information System in China (HIS) (66.56%), and picture archiving and communication system (40.26%) [12,13]. Similarly, hospitals and healthcare providers in the US are implementing EHRs rapidly in response to the American Recovery and Reinvestment Act of 2009 [14,15].

Peking University hospitals’ efforts to implement EHRs are timely and concurrent with similar initiatives in developed countries. As of 2006, there were 112 countries participating in the World Health Organization eHealth survey (as documented in Building Foundations for eHealth: Progress of Member States). At that time, five countries were approaching universal adoption of EHRs – Australia, Denmark, the Netherlands, Norway, and New Zealand. Three countries – United States (US), United Kingdom, and Germany – had made substantial progress; Japan and Canada had begun implementation efforts [16].

Among these countries, the US has adopted a standard for EHR functionality. Included in recent health care reform legislation, the US government offered financial incentives for hospitals and physician practices to implement EHRs that meet specified criteria [17]. These criteria, referred to as Meaningful Use (MU) objectives, are intended to promote improvements in clinical quality and safety indicators and continuous quality improvement. Hospital EHR MU phase one objectives consist of 14 Core requirements, plus ten optional (Menu) requirements. In general, all Core objectives and five of the Menu objectives must be met. An example of a Meaningful Use objective is to “maintain an up-to-date problem list of current and active diagnoses” [18].

The MU objectives incorporate valuable information about what EHR functionality is important for improved patient health and health care delivery efficiency in the US. While the organization of the health care systems of China and the US differ, physicians in both countries provide direct patient care and document in the EHR. As the US has substantial EHR implementations and China has begun EHR implementation efforts, this study compared MU objectives to the EHR functionality of two leading hospitals in China. This study was intended to provide valuable information about important EHR functionality to informaticians in the US and China. This study was designed to inform future EHR adoption and policy in China as well as informatics research in the US. We anticipated that informaticians in the US and China would find it useful to learn what MU objectives matched the EHR functionality of China’s leading hospitals’ EHRs and which MU objectives were not currently supported in these EHRs. Informaticians in China may use this information to develop standard EHR functionality criteria for EHRs in anticipation of increased EHR adoption. Furthermore, this study has the potential to stimulate more comprehensive informatics research. Informaticians in the US and elsewhere in the world may use this information to assess the generalizability of MU criteria beyond the US. In addition, describing the functionality of China’s hospitals’ EHRs using MU as a standard allows for comparability with US hospitals’ EHRs in future informatics research. This study will be among the first evaluation studies of EHRs in China’s hospitals and will add to the growing body of literature on MU.

## Methods

The study design was qualitative and descriptive using qualitative data collection and observation of physician EHR users, post-EHR implementation. The study design is similar to that used to assess MU functionality of a home care EHR [19]. Unlike the MU home care EHR study, software documentation from the EHR provider was not available for analysis. It should be noted that this study does not intend to compare the EHR functional requirements between the US and China as China does not have a certification program to date to standardize the functional offerings of EHR products.

A second dissimilarity involved the language challenges involved in conducting the research. One principal investigator (PS) was experienced in the study methodology; however, neither read nor spoke Chinese. The second principal investigator (JL) was fluent in both Chinese and English. JL translated what was observed and spoken where needed. To contribute toward a sustained informatics research educational effort in China, the study team also included one health informatics student (PG) from Peking University’s Center for Medical Informatics. The student read and spoke Chinese and English. The student researcher, under the supervision of JL, translated MU objectives from English to Chinese and participated in the observations and analysis. Both Institutional Review Boards of Peking University Medical College Hospitals and Drexel University approved the study.

### Setting

The study took place in the following two hospitals in Beijing. Chinese hospitals are classified by the Ministry of Health (MOH) as primary, secondary, and tertiary hospitals. Each category has three ranks (class A, class B, and class C) which are based on various institutional characteristics (e.g., size, staff, facility, quality). Tertiary class A is the best; both hospitals were in this class. The study sample consisted of one general hospital and one specialty hospital. The following provides a description of the hospitals and identifies their respective EHRs.

1. Peking University First Hospital (PUFH) was established in 1915. It is a comprehensive teaching hospital with 1,500 beds and 60 wards. Ambulatory visits are 7,000 per day; total hospital admissions are 45,000 per year and 20,000 surgeries are performed each year. The first hospital began implementing an EHR in 2010 using a commercial vendor. The computerized provider order entry (CPOE) system was scheduled to be implemented the week following the study.

2. Beijing Cancer Hospital (BCH) was established in 1976 as a specialty hospital. The hospital has 700 beds, 26 wards, and 13 ancillary departments. Annually, the hospital has 280,000 outpatient visits and 23,000 admissions. BCH does not treat children. The hospital implemented an EHR in 2006 using a second commercial vendor.

To better understand the clinical setting in China, the following is a general description of hospital information systems and how physicians at the hospitals use an EHR. Similar to the US, a hospital obtains clinical information systems from multiple vendors. Usually the EHR vendor differs from the Health Information System (HIS) vendor. The HIS system consists of CPOE, clinical decision support (CDSS), and pharmacy systems. Departmental information systems include laboratory, radiology, and picture archiving and communication systems. In China, the term EMR (electronic medical record) refers to the record of patient episodes in one hospital. The historic, narrower definition of EMR referred to physician documentation of patient information only. Currently, a broader definition of EMR approaches the US term EHR. In contrast, the term EHR in China refers to the computerized patient longitudinal health care record across care settings. EHRs are maintained by health departments and MOH has planned that patient clinical information is to be shared via regional health exchanges at national, provincial and metropolitan levels. Until now about 16 provinces and regions have started to implement these exchanges.

As for inpatient workflow, a patient first sees an outpatient department physician or surgeon who decides if the patient is to be hospitalized and, if so, writes an order for hospitalization. The patient usually waits until a bed is available. When the patient is informed there is bed available, he/she arrives at the hospital, usually with his/her handwritten ambulatory record. The patient first goes to the hospitalization department to complete their registration by providing demographic information and billing information. The patient next goes to the specified department and ward. In the ward, a nurse will assign a bed and the physician. (The latter will be discussed with the attending physician who is in charge of the ward). The nurse then takes the patient’s vital signs and informs the assigned physician who will care for the patient during the entire hospitalization.

The hospital physician is staff at the hospital. Physicians usually rotate between an ambulatory/outpatient department and the related inpatient department. The physician in charge, usually a resident, will: (1) perform the physical examination; (2) issue the initial treatment plan by placing orders in the CPOE; (3) write the admission note which is usually 3 to 5 pages long and completed within 24 hours following patient admission; and (4) document the first progress note which is required to be completed within 8 hours following admission. Also at the start of the patient stay or during the hospitalization, patient consent forms are printed to obtain patient signatures due to the absence of electronic signature functionality in China. Usually during each hospitalization day, the physician will see the patient and prescribe medications, order tests and procedures, or write a new progress note. The physician is required to write at least one progress note every three days or more if there are changes, until the patient is discharged with a discharge summary. Referrals to specialists tend to be less common than in the US.

**Figure 1:**
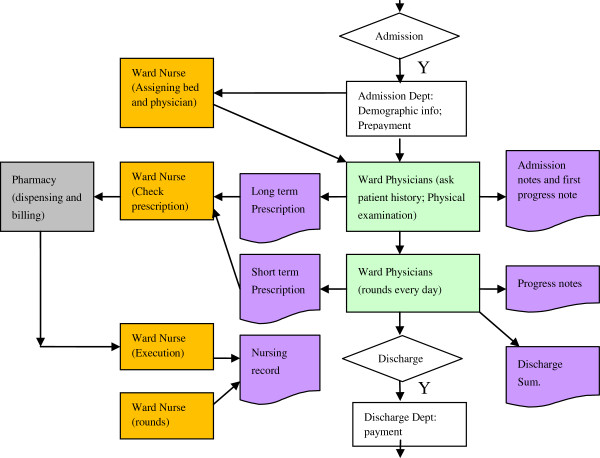
Chart of a typical inpatient workflow in China.

At the conclusion of the hospitalization, the medical record is archived and sent to the patient record department for storage and future inquiries. The archived complete medical record includes the admission note, first progress note, subsequent progress notes, discharge summary, prescription and lab results, as well as nursing notes. To comply with external reporting, a report is usually generated and provided to the hospital medical affairs/quality department. This department reviews, supplements, and submits the report to the external authority. Figure 1 illustrates a typical inpatient workflow.

### Observation/data collection

Prior to visiting each hospital, the team met to discuss each MU objective and its interpretation in the context of the health care system in China. For example, the demographic concept “race” is not used in China; the equivalent concept is “nationality”. Similarly, the term “preferred language” is not relevant because, although dialects are spoken, the Mandarin Chinese language is universally spoken.

At each hospital, the research team asked one physician to demonstrate how he used the EMR to document patient demographics and patient care. The researcher, JL, consented the physician, communicated with the physician, and translated for the researcher, PS, who documented in field notes. As the physician demonstrated functionality, the student researcher, PG, recorded the functionality on the MU objectives checklist which was written in both English and Chinese. After the physician had demonstrated the EMR, the physician was asked about MU functionality that had not been demonstrated. Following each hospital visit, the research team reviewed the findings and discussed whether there were plausible explanations for MU functionality not observed. JL contacted each hospital via telephone and inquired about functionality specific to the information technology (IT) department (e.g., Core #14-protection of health information) or hospital administration (e.g., Menu #8-submit data to immunization registries). JL followed up with PUFH, as CPOE-related functionality had not been identified during the observation phase of the research, but was implemented later. JL also contacted the IT department of each hospital to ask whether any functionality not observed was available in the EMR.

## Results

### Functionality of PUFH EMR

The PUFH EMR included less than half of the 14 Core Meaningful Use Objectives functionality, as shown in Table 1. The following functionality was not available in the EMR:

1. # 2: Implement drug-allergy interaction checks (drug-drug interaction checks are implemented; in PUFH the medication allergy list is unstructured text and not available as structured data as per MU) 2. # 3: Maintain an up-to-date problem list of current and active diagnoses (abnormal signs and symptoms of a definite diagnosis are not maintained)

2. # 5: Maintain active medication allergy list

3. # 6 (A): Preferred language; # 6 (C) Race

4. # 7: record all vital signs but do not chart changes (C): Blood pressure (recorded as free text, not as structured data); # 7 (D): BMI (recorded only for patients viewed as obese)

5. # 8: Smoking status (recorded as free text, not as structured data)

6. # 9: Report hospital clinical quality measures (outside the hospital)

7. # 10: Implement 1 CDSS (Clinical Decision Support System) rule

8. # 11: Provide patients with electronic copy of their health information (paper copy provided)

9. # 12: Provide patients with electronic copy of their discharge instructions (paper copy provided)

10. #13: Exchange key clinical information (cannot be shared among providers however hospitals are required to send case summary to MOH electronically)

**Table 1:** Comparison of EHR functionality of two leading hospitals in China to US EHR meaningful use objectives

**Objective**	**Peking University first hospital**		**Beijing cancer hospital**	
	**Observed**	**Available**	**Observed**	**Available**
Eligible Hospital and CAH Core Objectives				
(1) Use CPOE for medication orders directly entered by any licensed healthcare professional who can enter orders into the medical record per State, local, and professional guidelines	N	Y	Y	Y
(2) Implement drug-drug and drug-allergy interaction checks	N	Drug-drug (yes); drug-allergy (No)	N	Drug-drug (yes); drug-allergy (No)
(3) Maintain an up-to-date problem list of current and active diagnoses	N	Problem list (No); Diagnoses (Yes)	N	Problem list (No); Diagnoses (Yes)
(4) Maintain active medication list	N	Y	Y	Y
(5) Maintain active medication allergy list	N	N	Y	Y
(6) Record all of the following demographics:				
(A) Preferred language	N	N	N	N
(B) Gender	Y	Y	Y	Y
(C) Race	N	N	N	N
(D) Ethnicity	Y	Y	Y	Y
(E) Date of birth	Y	Y	Y	Y
(F) Date and preliminary cause of death in the event of mortality in the eligible hospital or CAH	Y	Y	Y	Y
(7) Record and chart changes in the following vital signs:				
(A) Height	N (record but no charting)	N (record but no charting)	N (record but no charting)	N (record but no charting)
(B) Weight	N (record but no charting)	N (record but no charting)	N (record but no charting)	N (record but no charting)
(C) Blood pressure	N (record but no charting)	N (record but no charting)	N (record but no charting)	N (record but no charting)
(D) Calculate and display body mass index (BMI)	N (only calculated for endocrinological department patients)	N	N (only for obese patients)	N
(E) Plot and display growth chart for children 2 to 20 years, including BMI	N	N	Not applicable	N
(8) Record smoking for patients 13 years old or older	N	N	Y	Y
(9) Report hospital clinical quality measures to CMS or, in the case of Medicaid eligible hospitals, the States	N	N	Y	Y
(10) Implement one clinical decision support rule related to a high priority hospital condition along with the ability to track compliance with that rule	N	N	N	N
(11) Provide patients with an electronic copy of their health information (including diagnostic test results, problem list, medication lists, medication allergies, discharge summary, procedures), upon request	N	N	N	N
(12) Provide patients with an electronic copy of their discharge instructions at time of discharge, upon request	N	N	N	N
(13) Capability to exchange key clinical information (for example, problem list, medication list, medication allergies, and diagnostic test results), among providers of care and patient authorized entities electronically	N	N	N	N
(14) Protect electronic health information created or maintained by the certified EHR technology through the implementation of appropriate technical capabilities	Y	Y	Y	Y
Eligible Hospital and CAH Menu Set Objectives				
(1) Implement drug formulary checks	N	Y	Y	Y
(2) Record advance directives for patient 65 years old or older	N	N	N	N
(3) Incorporate clinical lab-test results into EHR as structured data	Y	Y	Y	Y
(4) Generate lists of patients by specific conditions to use for quality improvement, reduction of disparities, research, or outreach	Y	Y	Y	Y
(5) Use certified EHR technology to identify patient-specific education resources and provide those resources to the patient if appropriate	N	N	N	N
(6) The eligible hospital or CAH who receives a patient from another setting of care or provider of care or believes an encounter is relevant should perform medication reconciliation	N	N	N	N
(7) The eligible hospital or CAH that transitions their patient to another setting of care or provider of care or refers their patient to another provider of care should provide summary care record for each transition of care or referral	Y	Y	Y	Y
(8) Capability to submit electronic data to immunization registries or immunization information systems and actual submission according to applicable law and practice	N	N	Not applicable.	Y
(9) Capability to submit electronic data on reportable (as required by State or local law) lab results to public health agencies and actual submission according to applicable law and practice	Y	Y	Y	Y
(10) Capability to submit electronic syndromic surveillance data to public health agencies and actual submission according to applicable law and practice	N	N	N	N

Of the 10 Menu MU Objectives, the following functionalities were not available at PUFH:

1. # 2: Record advance directives (recorded as free text on the consent form which is not in the EHR)

2. # 5: Patient-specific education resources (provided orally)

3. # 6: Medication reconciliation

4. # 8: Submit electronic immunization data

5. # 10 Syndromic surveillance data

### Functionality of BCH EMR

The BCH EMR included half the core objectives as shown in Table 1. Objectives related to pediatrics (e.g., # 7 (E) growth chart) were not applicable. The following functionality were not available at BCH:

1. # 2: Drug-allergy interactions(in BCH the allergy information is structured in the admission note and not interfaced into CPOE)

2. # 3: Maintain an up-to-date problem list of current and active diagnoses (active diagnoses only are maintained)

3. # 6 (A): Preferred language; # 6 (C) Race

4. # 7: Record and chart changes in vital changes

5. # 10: Implement 1 CDSS rule

6. # 11: Provide patients with electronic copy of their health information (paper copy provided)

7. # 12: Provide patients with electronic copy of their discharge instructions (paper copy provided)

8. #13: Exchange key clinical information

Similar to PUFH, of the 10 Menu Objectives, the following functionality were not available at BCH:

1. # 2: Record advance directives (recorded as free text on the consent form which is not in the EHR)

2. # 5: Patient-specific education resources (provided orally)

3. # 6: Medication reconciliation

4. # 10: Syndromic surveillance data

### Other findings

A number of observations from the study hospitals were of note. First, physicians documented all the patient clinical information that was documented, and no information was dictated. Second, the patient’s occupation was included in the documented patient demographic information. Third, physicians in both hospitals recorded current diagnoses using structured text. In addition, PUFH physicians used ICD-10 codes. We also learned that CDSS was not implemented at BCH because physicians were opposed to it. Fifth, both EMRs had functionality that enabled physicians to generate lists of patients by diagnosis. The discharge summary served as the summary care record. Neither hospital automatically generated a summary care record. Based on observation, the researcher (JL) identified eight sections of the discharge summary: Demographic, Chief complaints, Present history of disease, Admission diagnosis, Progress summary in hospital, Current status, Discharge diagnosis, and Discharge instructions/orders.

## Discussions and conclusions

This study is the first known assessment that compared hospital EMRs in China to the current US EHR standard, Meaningful Use objectives. This study sheds light on important US hospital EHR functionality that physicians in China actually use. To describe hospital EMRs in China, we used MU objectives because they were a de-facto national standard for necessary functionality for EHRs that listed discrete, specific US EHR functionalities. The health system in China differs from that of the US health system, and hospitals in China operate differently from US hospitals. Therefore, we expected the EMRs implemented in hospitals in China to differ from US EHR MU functionality.

The study findings indicated that the two EMRs studied met about half of the MU objectives. We found differences in EHR/EMR functionality that reflected cultural differences as well as operational differences. Cultural differences included that the EMRs in China have no need to capture preferred language or race due to the perceived homogeneity of these characteristics. Also, the US process for advanced directives is not present in the clinical care process in China. Instead, advanced directives are inferred from consent forms; patients in the study hospitals must specifically consent to each procedure before it is begun. Each consent form must be signed by hand. There is no checkbox to note the presence of an advanced directive.

Comparing MU Objectives and EMR functionality of the study hospitals in China, we identified operational differences at a number of levels. At the physician level, physicians were required to maintain a diagnoses list rather than a problem list which included abnormal signs and symptoms. Abnormal signs and symptoms are listed only when their origins are unknown since Chinese physicians emphasize causes of diseases.

Also, we did not observe medication reconciliation as an EMR function because infrequent care transitions greatly reduced the need for this functionality. The physician referred to the patient’s ambulatory record which they themselves, or a physician in their department, wrote. Medication continuity is relatively easily maintained by the same/similar physicians in the same hospital. Also, unlike physicians in US hospitals, Chinese physicians documented most of the care themselves and did not dictate any content.

In addition, at the implementation level, we noted that in both hospitals drug-allergy interaction functionality was not implemented. Usually in China, the HIS vendor implements the CPOE system where physicians input medication orders. Physicians document allergies as free text in the EMR provided by EMR vendors. The CPOE and HIS systems do not share medication allergy data in part due to the absence of standards, or due to the fact that Chinese physicians do not think this is a significant issue.

Regarding discharge, in the observed hospitals, the discharge summary included the content contained in the US summary care record. Also, physicians provided patient-specific education resources orally, unlike US hospitals where nurses tend to educate the patient and provide printed patient educational material. Finally, at the organizational level above the hospital, EMRs do not have the capability to support data exchange, external reporting, or syndromic surveillance.

Of the MU objectives that were not met, four may be easily implemented by vendors in China if they are required by the hospitals. For example, neither hospital provided patients with an electronic version of health information or education materials. Also, smoking status is implemented as free text rather than structured data. Plus vital signs are recorded but not charted.

The study hospitals in China had two notable EMR functionalities. First, similar to hospitals in other countries and unlike most US hospitals, diagnoses were coded using ICD-10 codes. Either the physician or medical record department staff documented ICD-10 codes when records were archived. Second, physicians recorded the patient’s occupation. While some states in the US collect occupation information using standardized occupation codes, recent national efforts have been focused on documenting occupation in the health record using standardized codes [20].

We also found similarities between the US hospitals and the study hospitals in China. In both countries, MU Objectives not met included that hospitals were unable to exchange patient clinical information electronically with other sites that provided care. Instead, the study hospitals gave patients paper copies of their clinical information. In addition, in PUFH it was not unexpected that one CDSS rule had not been implemented. CPOE was newly implemented and, as in US hospitals, CDSS tended to be implemented after CPOE implementation. Of particular interest was BCH’s not implementing CDSS based on physicians’ objections.

It was also very interesting that there was a lack of uniformity of the EMR functionality in these two hospitals. For example, related to the MU Core objectives, there was similarity in recording demographics and diagnoses, but not vital signs or smoking status. This lack of uniformity between the EMRs in two of the leading hospitals in China suggests the need for a national EMR standard similar to Meaningful Use.

Finally, in this study, the hospitals in China chosen to be assessed were not representative of all hospitals in China, as they were two top-tier tertiary hospitals in Beijing that were early adopters of EMRs. It is possible that the EMRs studied may not be representative of all EMRs implemented in China. While the study hospitals had recently implemented their EMRs, it is possible that other hospitals may have EMR systems with more functionality that matched MU objectives.

Due to China’s 2009 health reform initiative’s strong policy support, there has been rapid HIT adoption, including EMR adoption, by hospitals. In contrast, the US financial incentives for hospitals to implement EHRs that meet MU objectives may be less effective in encouraging EHR adoption as compared to policy support in China. While China is not viewing U.S. MU objectives as a gold standard for comparison, the MU approach and objectives can provide China a model for standardizing and evaluating EMR adoption. China has issued more than 100 HIT standards during 2010–2011 but lack standards like U.S. MU that are able to test and assess the level of EMR adoption. In the near future, China should implement not only an EMR certification program, which guarantees the required functionalities of EMR standards, but also a U.S. MU-like standards and certification program to ensure the quality of EMR adoption rather than just raise the rate of EMR adoption.

## Competing interests

The authors declare that they have no competing interests.

## Authors’ contributions

JL and PS developed the conceptual framework and research protocol for the study. PS drafted the manuscript and JL made major revisions. Master student PG conducted the onsite translation and data collection. QM and JZ provided comments and made revisions to the manuscript. All authors read and approved the final manuscript.

## Pre-publication history

The pre-publication history for this paper can be accessed here:

http://www.biomedcentral.com/1472-6947/13/96/prepub

## References

[B1] Ministry of Health, China Opinions of the CPC central committee and the state council on deepening the health care system reformhttp://shs.ndrc.gov.cn/ygjd/ygwj/t20090408_271138.htm. Accessed October 3, 2012

[B2] Meng Q Xu L Zhang Y Qian J Cai M Xin Y Gao J Xu K Boerma JT Barber SL Trends in access to health services and financial protection in China between 2003 and 2011: a cross-sectional study Lancet 2012 379 805 814 10.1016/S0140-6736(12)60278-5 22386034

[B3] Sinclair JAC China’s Healthcare reform The China Business Review 2009 36 52 32 35

[B4] Blumenthal D Glaser JP Information technology comes to medicine N Engl J Med 2007 356 2527 2534 10.1056/NEJMhpr066212 17568035

[B5] General Office of MOH Notice of the General Office of the Ministry of Health to solicit, comments of “the basic architecture and data standards of electronic medical records (draft)” http://www.moh.gov.cn/mohbgt/s6718/200912/45414.shtml. Accessed September 12, 2013

[B6] Medical Affairs Office of MOH of China Notice of the Ministry of Health on the issuance of “ the basic regulations of electronic medical records(Trial)” http://www.moh.gov.cn/publicfiles/business/htmlfiles/mohyzs/s3585/201003/46174.htm. Accessed October 23, 2012

[B7] Medical Affairs Office of MOH of China Notice of the Ministry of Health on the issuance of the functional specifications of electronic medical record system (Trial) http://www.moh.gov.cn/mohyzs/s3585/201012/50229.shtml. Accessed September 12, 2013

[B8] Center for Statistics and Informatics of MOH of China The opinion letter of Center of Statistics and Informatics of MOH to seek public comments on “Technical specifications of hospital information platform based on the electronic medical records, (draft)” [http://www.moh.gov.cn/mohwsbwstjxxzx/s7968/201103/51079.shtml]. Accessed September 12, 2013

[B9] Center for Statistics and Informatics of MOH of China The opinion letter of Center of Statistics and Informatics of MOH to seek public comments on “Technical specifications of regional health information platform technology based on the electronic health record of residents (draft)” http://www.moh.gov.cn/mohbgt/s6693/200906/41031.shtml, Accessed October 23, 2012

[B10] General Office of MOH of China The notice of the General Office of the Ministry of Health on the promotion of pilot program of building electronic medical records as the core hospital informationhttp://wenku.baidu.com/view/e098b6020740be1e650e9a9f.html. Accessed September 12, 2013.

[B11] General Office of MOH of China The notice of the General Office of the Ministry of Health on the issuance of the second batch of electronic medical records pilot cities and pilot hospitalshttp://wenku.baidu.com/view/e6226d0ebb68a98271fefae6.html. Accessed September 12, 2013

[B12] China Hospital Information Management Association The white paper on China’s hospital information systems 2008 http://wenku.baidu.com/view/beb7c06eaf1ffc4ffe47ac90.html. Accessed September 12, 201323983638

[B13] China Hospital Information Management Association Survey of health information technology adoption status among Chinese hospitals, 2011–2012 http://www.chima.org.cn/index.php?m=content&c=index&a=show&catid=10&id=145; accessed August 25, 2013

[B14] Tagalicod R Anthony R Kahn J Medicare & Medicaid EHR incentive programs 2012 http://www.healthit.gov/providers-professionals/centers-medicare-medicaid-services. Accessed September 12, 201324030634

[B15] Blumenthal D Stimulating the adoption of health information technology N Engl J Med 2009 360 1477 1479 10.1056/NEJMp0901592 19321856

[B16] Jha A Blumenthal D International adoption of electronic health records, health information technology in the United States: where we stand ONC and RWJF 2008 7 104 142

[B17] the 111th Congress American recovery and reinvestment act ARRA 2009 2009 PL 111 PL 115

[B18] Office of the National Coordinator for Health Information Technology (ONC), Department of Health and Human Services: Health Information Technology Initial Set of standards, implementation specifications, and certification criteria for electronic health record technology Fed Reg 2010 45 170 20677416

[B19] Sockolow PS Adelsberger M Bossone C Bowles KH Identifying certification criteriafor home care electronic health record meaningful use AMIA Annu Symp Proc 2011 2011 1280 1289 22195189 PMC3243187

[B20] Taylor JA A call to collect industry and occupation codes in healthcare data; White paper 2011 http://publichealth.drexel.edu/SiteData/docs/ACalltoCol/a18fbd78dd30d9b4/A%20Call%20to%20Collect%20Industry%20and%20Occupation%20Codes%202012%20updated%202.pdf; accessed August 25, 2013

